# The morbidity of systemic steroids: analyzing its use in bullous pemphigoid^☆^

**DOI:** 10.1016/j.abd.2025.501266

**Published:** 2026-01-07

**Authors:** Lula María Nieto-Benito, Ricardo Suárez-Fernández

**Affiliations:** a Department of Dermatology, Hospital Central de la Defensa Gomez Ulla, Madrid, Spain; b Department of Dermatology, Hospital General Universitario Gregorio Marañón, Madrid, Spain

Dear Editor,

Bullous Pemphigoid (BP) is the most common autoimmune bullous disease.^1^ High-potency topical corticosteroids are accepted as first-line treatment in BP.^1^, ^2^ However, due to the chronic nature of the disease, which is often characterized by multiple relapses, or in moderate to severe/extensive cases, when topical therapies may be insufficient, systemic treatment is usually required to achieve disease control. Systemic steroids are typically prescribed after the failure of topical strategies^1^, ^2^ and are considered a second-line treatment according to most BP guidelines (doses of 0.5‒1 mg/kg/day prednisone).^1^, ^2^ It is estimated that systemic steroids are used in 70%‒80% of BP patients.^3^

However, their use has been classically associated with higher mortality and an increased risk of side-effects, particularly in elderly patients, who represent the majority of BP cases.^3^, ^4^ The aim of this study was to analyze the management of BP in our setting, with a specific focus on the outcomes of patients treated with systemic steroids.

A retrospective observational study was conducted, including all patients diagnosed with BP who were attended in our department between the year 2000 and the first semester of 2020. Diagnosis of BP was based on the updated diagnostic criteria.^1^ All cases included had at least three out of the four criteria: clinical, histopathological, serological (including Indirect Immunofluorescence [IIF] and/or Enzyme-Linked Immunosorbent Assay [ELISA] and Direct Immunofluorescence [DIF]). Epidemiological (including comorbidities that were present at the time of BP diagnosis (neurological, neoplastic diseases, and diabetes mellitus type 2), clinical, immunological, histopathological, and therapeutic characteristics of all patients were recorded, including dosage, duration, lines of treatment, definitive treatment, need for hospitalization, and any adverse events if present.

AntiBP180 IgG antibodies detected were directed against the Noncollagenous 16A domain (NC16A) by BP180-NC16A ELISA assay.

A commercially available ELISA kit was used that includes the detection of IgG autoantibodies against desmoglein 1, desmoglein 3, BP180, and type VII collagen. Full-length BP180 autoantibodies detection was not performed.

Categorical variables were expressed as total numbers with percentages, and continuous variables were expressed as means, with standard deviation in symmetric distributions or medians and interquartile ranges in asymmetric variables. Demographic, clinical, histopathological, and serological characteristics were compared between deceased and non-deceased groups, using either the Chi-Square test (or Fisher’s exact test when necessary) or Student’s *t*-test (or Mann-Whitney *U*-test/Wilcoxon W-test when necessary). The bilateral F-test was used for the equal variance test. Statistical analysis was performed with Stata (version 16 StataCorp, College Station, Texas, USA).

A total of 257 patients were included; 154 were male (59.9%) with a global mean age (±SD) at diagnosis of 80.5-years (±10.4). In regard to comorbidities, diabetes mellitus type 2 was present in 106 cases (41.2%) and neurological and oncological diseases in 102 (39.7%) and 47 (18.3%) patients, respectively.

We classified BP in four groups depending on the Body Surface Area (BSA) affected: generalized (>50% BSA) in 81 patients (31.5%); trunk and extremities (< 50%, affecting extremities and trunk) in 122 (47.5%), trunk (<50%, affecting only trunk) in 13 (5.1%), extremities (<50%, affecting only extremities) in 39 (15.2%) and “other” (when not corresponding to prior criteria) in 2 cases (0.8%). Scalp and mucosal involvement were present in 22 and 16 patients, respectively. Due to incomplete information in the clinical records, we were unable to assess the presence of inflammatory versus non-inflammatory phenotypes or calculate any disease severity scores (BPDAI or IGA).

All skin biopsies showed a subepidermal blister with an eosinophilic inflammatory infiltrate and, except for 11 patients (4.3%), DIF revealed immunofluorescence patterns compatible with BP. In terms of serology, antiBP180 antibodies were found positive (>20 U/mL) in all cases in which these autoantibodies were tested (170 patients; 66.1%); the mean concentration (±SD) was 49.13 (±53.63).

Of the 257 BP patients studied, all received topical treatment; 41 (16%) achieved complete remission after high/very high-potency topical corticosteroids as first-line treatment. Systemic steroids were prescribed in 209 cases. In 125 patients (48.6%) of the study cohort, systemic corticosteroids (with or without topical treatment) were the definitive treatment.

However, 91 patients required additional interventions to control the disease. In 234 cases (92.3%), remission was achieved with three or fewer lines of treatment. Among those receiving systemic steroid treatment, in 85.1% of cases, doses lower than 0.5 mg/kg/day (0.3‒0.4 mg/kg/day) were prescribed. Mean steroid treatment duration was 6.33 (±1.77 SD) months (Table 1). None of the patients were treated with plasma exchange, intravenous immunoglobulin, or steroid pulse therapy.

**Table 1:** Therapeutic and management characteristics of BP patients included in the study.

	N = 257	Definitive treatment, n (%)	Definitive treatment, n (%)
Treatment	n (%)	N = 257	N = Total of patients that received it
Topical corticosteroid	257 (100)	41 (16)	41 (16)
Systemic steroid (prednisone)	209 (81.3)	125 (48.6)	125 (59.8)
<0.5 mg/kg/day	178 (69.2)		
>0.5 mg/kg/day	28 (10.9)		
Azathioprine	63 (24.5)	50 (19.5)	50 (79.4)
Tetracyclines	29 (11.3)	12 (4.7)	12 (41.4)
Associated to nicotinamide	19 (7.4)	8 (3.1)	8 (42.1)
Methotrexate	21 (8.2)	16 (6.2)	16 (76.2)
Dapsone	11 (4.3)	7 (2.7)	7 (63.6)
Rituximab	6 (1.9)	6 (1.9)	6 (100)
**Treatment duration of systemic steroids (prednisone) (months)**			
Mean (±DS)	6.33 ± 1.77		
Median (p25‒p75)	6 (3‒9)		
**Number of lines of treatments employed to achieve disease control [N = 257, n (%)]**			
1	41 (16)		
2	127 (49.4)		
3	69 (26.8)		
4	14 (5.4)		
5	3 (1.2)		
6	3 (1.2)		
**Number of flares/recurrences [N = 257, n (%)]**			
0	198 (77)		
1	46 (17.9)		
2	9 (3.5)		
3	3 (1.2)		
4	1 (0.4)		
**Hospitalization [N = 257 n (%)]**			
Sí	93 (36.2)		
No	164 (63.8)		
**Hospitalization time (days)**			
Mean ± DS	5.91 ± 9.66		
Median (p25‒p75)	5 (2‒9)		

Definitive treatment was defined by the therapeutic strategy that led to achiev} complete remission. Percentages in the second column refer to the global study cohort (n = 257); in the third column, only patients who received the referred treatment were considered.

By the end of the study period, 155 BP cases had died. There were no significant differences in terms of age, sex, neoplastic comorbidities, or BSA between deceased and non-deceased patients (Table 2).

**Table 2:** Analysis of the demographics, comorbidities, serological, therapeutics and hospitalization outcomes in deceased and non-deceased BP patients included in the study.

	Deceased	Non-deceased	p
	N = 155, n (%)	N = 102, n (%)	
**Mean age** (±SD) (years)	83.78 (± 8.25)	85.48 (± 11.39)	0.378
**Sex**			0.284
Female	58 (37.4)	45 (44.1)	
Male	97 (62.6)	57 (55.9)	
**Neurological comorbidities**	78 (50.3)	24 (23.5)	**0.000**
Senile dementia	22 (14.2)	4 (3.9)	
Alzheimer’s Disease	19 (12.3)	7 (6.9)	
Vascular dementia	18 (11.6)	8 (7.8)	
Parkinson’s disease	14 (9)	3 (2.9)	
Amyotrophic lateral sclerosis	3 (1.9)	0	
Multiple sclerosis	0	2 (2)	
Lewy body dementia	2 (1.3)	0	
**Neoplastic comorbidities**	29 (18.7)	18 (17.6)	0.839
**Diabetes mellitus type 2**	62 (40)	44 (43.1)	0.617
**Body Surface Area (BSA)**			0.532
Generalized (>50%)	49 (31.5)	32 (31.4)	
Trunk and extremities (<50%)	77 (49.6)	45 (44.1)	
Trunk (<50%)	6 (3.8)	7 (6.8)	
Extremities (<50%)	22 (14.1)	17 (16.7)	
Other	1 (1)	1 (1)	
**Anti-BP180 antibodies**			
Number of patients tested	102 (65.8)	78 (76.5)	0.358
Positivity (>20 U/mL)	56 (36.1)	59 (57.8)	0.692
Mean titer (±SD) (U/mL)	50.95 (±49.32)	41.93 (±56.48)	**0.050**
**Definitive treatment**			
Topical corticosteroids	155 (100)	102 (100)	‒
Systemic steroids (prednisone)	130 (83.9)	79 (68.6)	0.196
Doses of systemic steroids			0.118
<0.5 mg/kg/día	108 (69.7)	72 (70.6)	
>0.5 mg/kg/día	22 (14.2)	7 (6.9)	
Treatment duration of systemic steroids (months)			0.235
Mean (± DS)	7.45 (± 1.03)	6.02 (± 2.41)	
Median (p25‒p75)	6 (3‒9)	6 (3‒9)	
Mean steroid cumulative doses (mg/treatment/patient)	5369.54	4912.41	0.156
Azathioprine	34 (21.9)	29 (28.4)	0.236
Methotrexate	5 (3.2)	16 (15.7)	NS
Dapsone	1 (0.6)	10 (9.8)	NS
Tetracyclines [+ nicotinamide]	14 (9) [11 (7.1)]	15 (14.7) [8 (7.8)]	0.160
**Hospitalization**			
Need for hospitalization	62 (40)	31 (30.4)	0.117
Hospitalization time (days) [mean (±DS)]	5.58 (± 8.74)	6.47 (± 11.09)	0.891

However, neurological comorbidities were significantly more common in the group of deceased BP patients (50.3% vs. 23.5%, p = 0.000) (Table 2). No specific neurological disease, including vascular dementia, was more prevalent in the deceased group.

Although no differences were found in the detection rate of anti-BP180 autoantibodies, the concentration of these antibodies was higher in the group of patients who had deceased by the end of the study (p = 0.050) (Table 2).

Regarding therapeutic outcomes, there were no significant differences in definitive treatment, steroid doses, cumulative steroid doses or need for hospitalization (Table 2).

Additionally, higher steroid doses (>0.5 mg/kg/day) were not associated with a worse prognosis in terms of survival time (from BP diagnosis to death) compared to lower doses (median survival time: 5-years [95% CI 3.43‒6.58] for higher doses vs. 6-years [95% CI 4.04‒7.96] for lower doses). Mean survival time for other systemic treatments different from systemic corticosteroids found was 5-years (95% CI 3.02–6.98). No adverse events directly related to systemic steroids were observed.

Systemic corticosteroids have traditionally been linked to higher mortality and increased side effects, especially when employed at high doses (1 mg/kg/day).^5^, ^6^ Their use is generally recommended to be limited to younger patients, elevated BPDAI scores, and high concentrations of antiBP180 NC16A antibodies.^4^, ^5^, ^6^ Lower doses (0.1‒0.2 mg/kg) have also been associated with a higher risk of relapse.^3^, ^4^

However, in agreement with our findings, several studies have reported no increase in mortality in patients treated with systemic steroids.^3^, ^4^, ^7^, ^8^ When investigating whether early initiation of corticosteroid-sparing therapy in BP patients resulted in better outcomes than late or no sparing, the rate of complications and 1-year mortality did not differ significantly among therapeutic strategies.^7^

Moreover, mortality in BP patients has been associated with older ages, presence of multimorbidity, higher concentration of anti-BP180 and anti-BP230 antibodies, greater disease activity, and higher levels of systemic inflammation.^3^, ^8^, ^9^, ^10^

In conclusion, systemic corticosteroids, despite their classical association with increased mortality, may not necessarily elevate this risk. Doses of lower than 0.5 mg/kg/day of prednisone appear sufficient to control skin lesions in a significant number of patients while minimizing morbidity in BP patients. The advanced age of most BP patients complicates efforts to improve quality of life and prevent recurrences when immunosuppressive drugs are required. Although treatment should always balance benefits and risk, patients with BP must be treated appropriately, and therapeutics must be adapted to disease severity, in order to reduce systemic inflammation and avoid poor outcomes

## IRB approval status

This study was reviewed and approved by the ethics committee of the Hospital General Universitario Gregorio Marañón (CEIC). Approval from our ethics institutional board was obtained prior to the design and production of this paper. This study was carried out in accordance with the Declaration of Helsinki.

## ORCID ID

Lula María Nieto-Benito: 0000-0002-7609-2736

Ricardo Suárez-Fernández: 0000-0002-6065-1265

## Financial support

The authors have not received any financial support or funding for this project.

## Authors' contributions

Lula María Nieto Benito: Study concept and design; data collection, analysis, and interpretation of data; statistical analysis; writing of the manuscript; critical review of important intellectual content; critical review of the literature; final approval of the final version of the manuscript.

Ricardo Suárez Fernández: Study concept and design; final approval of the final version of the manuscript.

## Research data availability

The entire dataset supporting the results of this study was published in this article.

## Conflicts of interest

None declared.
